# A Review on Polymer and Lipid-Based Nanocarriers and Its Application to Nano-Pharmaceutical and Food-Based Systems

**DOI:** 10.3389/fnut.2021.783831

**Published:** 2021-12-01

**Authors:** Hongyun Lu, Shengliang Zhang, Jinling Wang, Qihe Chen

**Affiliations:** ^1^Department of Food Science and Nutrition, Zhejiang University, Hangzhou, China; ^2^School of Forestry, Northeast Forestry University, Harbin, China

**Keywords:** lipid-based nanocarriers, polymer-based nanocarriers, phytochemical, nanoformulation, drug and food application

## Abstract

Recently, owing to well-controlled release, enhanced distribution and increased permeability, nanocarriers used for alternative drug and food-delivery strategies have received increasingly attentions. Nanocarriers have attracted a large amount of interest as potential carriers of various bioactive molecules for multiple applications. Drug and food-based delivery *via* polymeric-based nanocarriers and lipid-based nanocarriers has been widely investigated. Nanocarriers, especially liposomes, are more and more widely used in the area of novel nano-pharmaceutical or food-based design. Herein, we aimed to discuss the recent advancement of different surface-engineered nanocarriers type, along with cutting-edge applications for food and nanomedicine and highlight the alternative of phytochemical as nanocarrier. Additionally, safety concern of nanocarriers was also highlighted.

## Introduction

There have been major breakthroughs in the field of nanotechnology, especially in the fields of materials science, electronics, photonics, supramolecular assembly, drug delivery, agriculture and food industries. In particular, the application of nanotechnology in medicine, usually termed nanomedicine, has provided a key impetus for the development of various types of drug-carrying nanomaterials (1, 2), such as liposomes, micelles, polymeric nanoparticles, albumin-based formulations (3). Typical nanomaterials possess several common characteristics: high surface-to-volume ratio, enhanced electrical conductivity, superparamagnetic behavior, spectral shift of optical absorption, and unique fluorescence properties. Nanomedicine is being taken as a promising way in drug delivery and diagnostics. To date, due to improved penetration and delivery of drugs into specific regions of skin, nanocarriers taken as alternative drug-delivery strategies have gained increasingly interest. A large number of studies have focused on nanocarriers as effective diagnostic or therapeutic tools for serious diseases, such as cancer, infectious or neurodegenerative diseases (4, 5). Besides, nanocarriers could not only improve the solubility of hydrophobic nutraceuticals more efficiently, but also they have almost no effect on the appearance of final food products, such as, drinks and beverages (6). Research has highlighted the complexity of the physicochemical and biochemical processes involved in the bioavailability of biocomponents, such as release, absorption, distribution, metabolism, and excretion, which contribute to increase the bioavailability of bioactive compounds used in food fortification (7).

**Figure 1:**
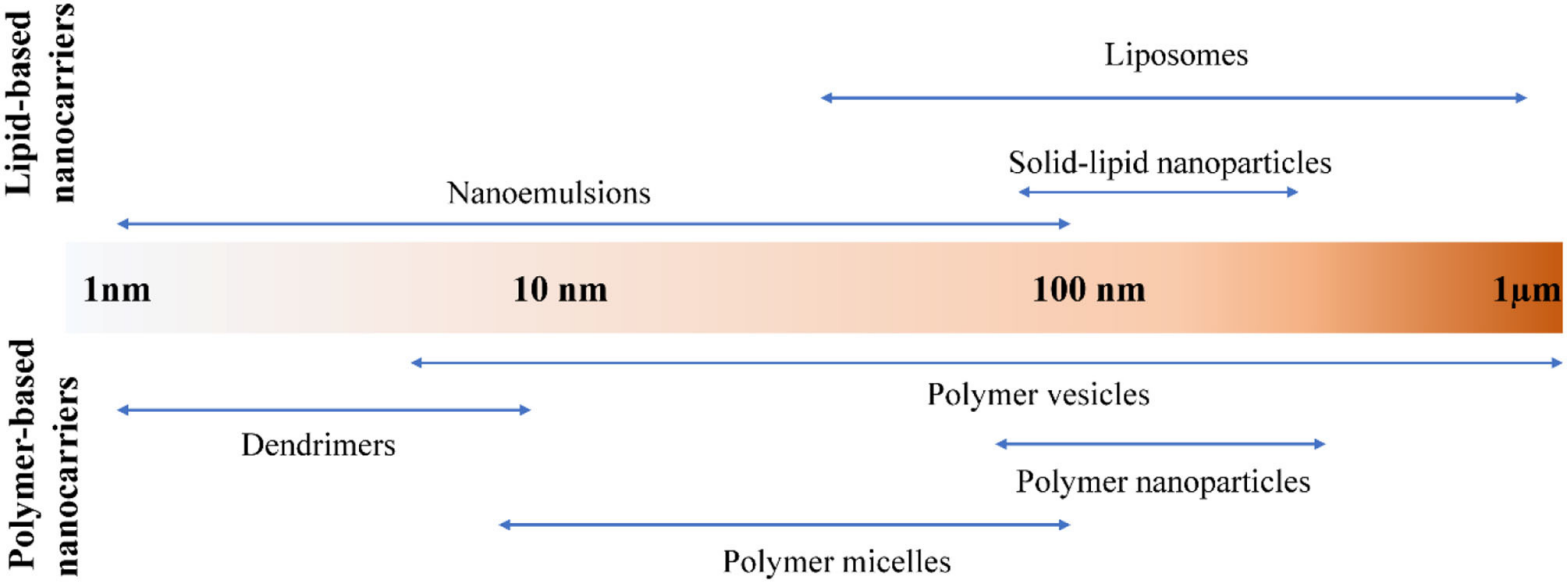
Lipid and polymer-based nanocarrier systems.

In general, nanocarriers are colloidal in size with diameters ranging from 1 to 1,000 nm (8). The nano-range size of medical nanocarrier is generally 1–100 nm. According to the different materials used to construct the carrier, it can be roughly divided into two types: lipid-based nanocarrier and polymer-based or dendritic nanocarrier. Among them, lipid-based nanocarriers can be divided into liposomes and solid-lipid nanoparticles. Nanocarriers based on polymers and dendritic branches are classified into dendritic branches, polymer micelles, polymer vesicles, nanoparticles and nano-colloids (8). Their particle size range is shown in Figure 1. In this review, we will have a brief overview of nanoencapsulation systems applicable to nanomedicine and food ingredients. And then, application of these nanocarriers for drug usage and different food bioactive components will be covered.

## Lipid-Based Nanocarriers

Lipid-based nanocarriers (LNs) are generally non-spherical in shape, which is either determined by the electrostatic interaction between polar/ionogenic phospholipid head and the solvent or due to non-polar lipid hydrocarbon moieties present in the solvent (9, 10). The unique physicochemical properties of LNs which in the form of liposomes or solid core lipid nanoparticles with excellent biocompatibility makes them candidates as carriers for drugs and food application. These LNs made of uniform lipid bilayers or solid cores can entrap various cytotoxic drugs. Hydrophilic drug will be trapped in the aqueous region, while the lipophilic drug will be captured in the lipid leaflets. Additionally, LNs carry drugs safely to the destination-tumor site, release it in a gradual manner, and are then degraded (9). Recently, research on lipid-based nanomaterials is blooming and main categories, including liposomes, solid lipid nanoparticles (SLNs), and nanostructured lipid carriers (NLCs) and nanoemulsions, have been receiving great attention in current research and clinical trials.

### Liposomes

Liposomes, the first phospholipid vesicle system developed in the 1960s (11), are composed of phospholipid bilayer similar to the plasma membrane of human cells. Therefore, liposomes have good biocompatibility and can promote drug diffusion across the plasma membrane (12). Liposomes can be designed as self-assembled vesicles comprising one or multiple concentric lipid bilayers that enclose an aqueous core (13). Liposomes, ranged from 20 nm to more than 1 μm, have the typical structure encapsulate with a hydrophobic bilayer and a hydrophilic core. Therefore, liposomes can hold and stabilize hydrophilic drugs in the hydrophilic core and encapsulate lipophilic drugs in lipid bilayer. Therefore, liposomes have good ability to carry both hydrophilic and hydrophobic drugs in the aqueous lumen and lipid bilayer, respectively, which contributing to the versatility of liposomes. In addition, liposome system also has the advantages of easy modification and targeting potential, which could be constructed with the surface modified with appropriated molecules (or ligands) to actively bind a target molecule of certain cells, system, or tissue (14). Since Doxil^®^ was approved by the FDA in 1995 as the first long-circulating liposome for cancer treatment, many chemicals have also been reported to be able to encapsulate liposomes. However, due to the limited bilayer space of liposomes, it is difficult to achieve high drug loading of hydrophobic drugs. It is necessary to strike a delicate balance between high drug loading and particle size distribution and stability of liposomes. To better improve clinical translation, further research is needed for targeted drug delivery by nano-carriers to reduce toxicity, enhance permeability and retention effects, and minimize the shielding effect of protein corona (15). Therefore, it is critical to optimize the composition, preparation methods and properties of the lipid bilayer.

### Solid-Lipid Nanoparticles (SLN)

Solid-lipid nanoparticles (SLN) were developed in the 1990s in order to combine the advantages of polymer nanocarriers, such as strong drug loading capacity, controllable drug delivery, good biocompatibility of lipid emulsions and improvement of drug bioavailability (16, 17). SLN can be prepared by a variety of technologies including heat or cold homogenization, which is easy to scale up production, has good preparation repeatability and does not require toxic organic solvents in the preparation process (16, 18). The main feature of SLN is that it contains lipids that remain solid at room temperature. Biocompatible substances such as triglycerides, fatty acids, steroids and biowaxes are often used to prepare SLN systems. Due to their small sizes and large surface area, SLN are suitable to be covered with functionalized ligands moieties, antibody and other functional group (19). SLNs can be orally administered as aqueous dispersions or in the dosage forms of capsules, tablets, and pellets (20). Among the different types of nanocarriers, SLN are at the forefront of the potential application in oral drug delivery systems (21). SLN have many advantages like easy manufacturing, the stability of pharmaceuticals, increased drug content, effective release of drug and high long-term stability. Additionally In terms of drug delivery, SLN system can efficiently encapsulate antitumor drugs and other substances with poor water-solubility due to its high lipid content (22).

### Nanostructured Lipid Carriers (NLCs)

Nanostructured lipid carriers (NLCs), also known as lipid-based formulations, have been broadly studied as drug delivery system. They have a solid matrix at room temperature and are considered superior to many other traditional lipid-based nanocarriers such as nanoemulsions, liposomes and SLNs due to their enhanced physical stability, improved drug loading capacity, and biocompatibility (23). To enhance biocompatibility and superior formulation properties, researchers modified SLN by replacing a fraction of solid lipids with liquid lipids to obtain nanostructured lipid carrier (NLC). Unlike SLN, the lipid matrix of NLC consisted of mixture of solid and liquid lipids with controlled levels that possess improved capacity of bioactive retention along with controlled release attributes (24). Compared to other lipid-based formulations, NLCs are biocompatible systems distinguished by a rigid morphology which contributes to unique properties (25). The wide variety of lipids used in topical lipid nanoparticulate formulations may be classified as fatty acids, waxes, steroids, partial glycerides, and triglycerides (26). Generally, NLC can be divided into three types: one is to use structurally different lipids to construct NLC; the other is to use amorphous lipids to construct NLC; the most common NLC system is composed of a mixture of solid lipids and liquid lipids (27, 28). Compared with solid lipids, drugs have a higher solubility in oil, thus common NLC has a stronger encapsulation ability for drugs than SLN (17). However, NLC has the disadvantage of difficult surface functionalization (29).

### Nanoemulsions

Nanoemulsions, referred to as dispersed systems with ≤100 nm droplets, are gaining importance in healthcare and cosmetics sectors as a result of the unique properties of nanosized droplets, such as high surface area (30). The small-sized droplet with a high surface area makes nanoemulsions important in many industries (31). High and low energy methods are used to prepare nanoemulsions, such as high-pressure homogenization, ultrasonication, phase inversion temperature and emulsion inversion point, bubble bursting method (32). Based on the components, Nanoemulsions can be categorized in three types, that is oil in water (O/W) type, water in oil (W/O) type, and bi-continuous/multiple emulsion where micro domains of oil and water phases are inter-dispersed (W/O/W and O/W/O) (33–36). Additionally, based on surface charge over the nano-droplets, nanoemulsions are categorized as neutral, anionic and cationic nanoemulsions (35). Digestible oils such as soybean oil, sesame oil, cottonseed oil or safflower oil are usually used to dissolve lipophiles (37). Compared to other nanocarriers, nanoemulsions are easy to prepare and do not necessarily require organic solvents/cosolvents, especially carriers prepared from edible oil with a low risk of toxicity. Based on the above advantages, nanoemulsions have been used to wrap paclitaxel, polyenetaxel, curcumin, retinoic acid and other anti-tumor drugs (38–42), and a large number of studies have reported using nanoemulsions to package essential oils, nutrients and other substances (43, 44).

**Figure 2:**
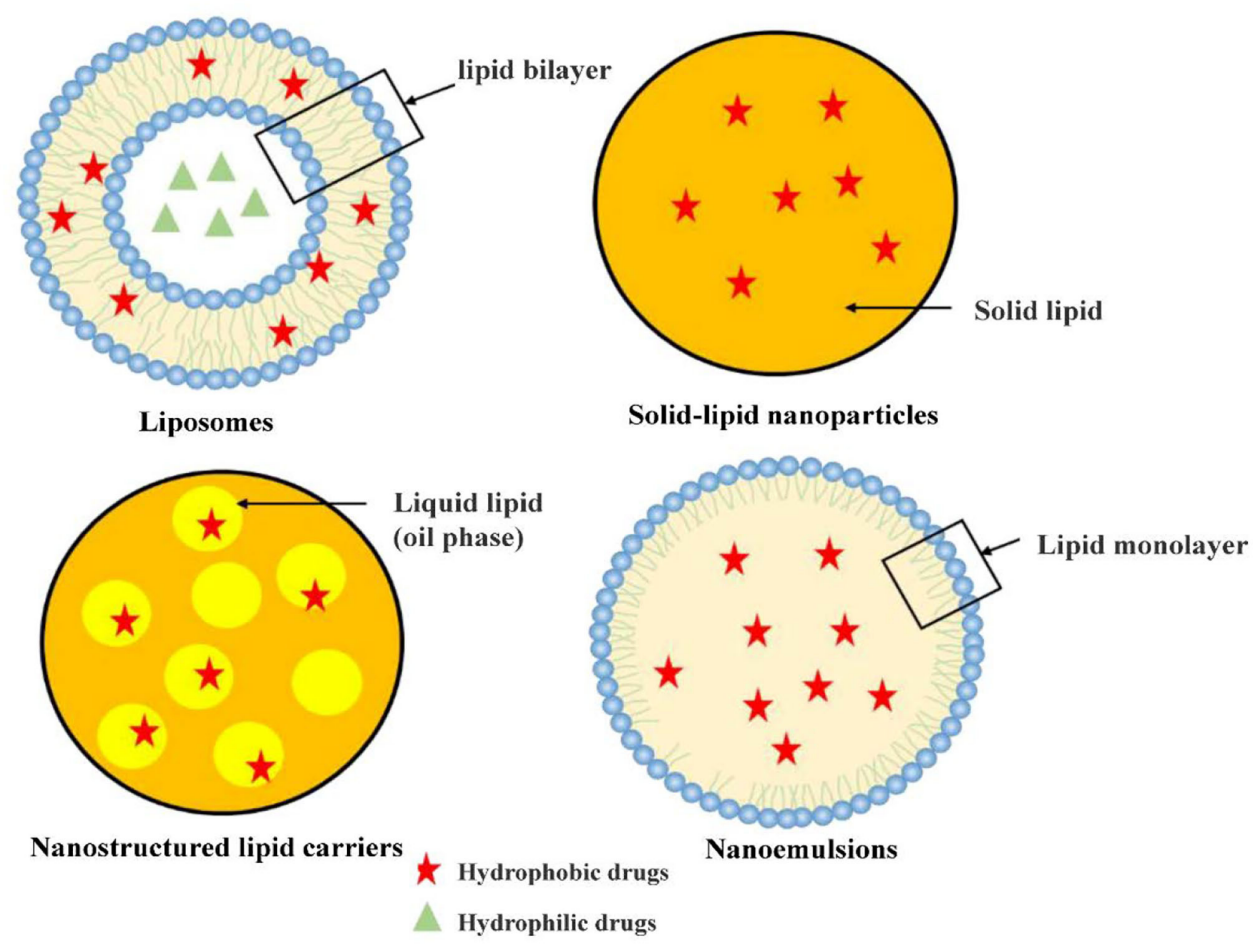
The classification of nanocarriers based on lipids or phospholipids.

Additionally, nanoemulsions have potential application in the food industry for the delivery of nutraceuticals, coloring and flavoring agents, and antimicrobials. The nanoemulsion formulations of active ingredients can be used for developing biodegradable coating and packaging films to enhance the quality, functional properties, nutritional value, and shelf life of foods. Considering nanoemulsions are susceptible to destabilization, essential oils (EOs) incorporated in nanoemulsions could be helpful to improved stability of nanoemulsion for high long-term stability food products (45). Nanoemulsion based edible nanocoatings containing flavor and coloring ingredients, antioxidants, enzymes, antimicrobials, and antibrowning agents can be used to coat foods such as meats, dairy products such as cheese, fresh produce, and fresh cuts including fruit and vegetables and confectionaries to improve their shelf life (45). Totally speaking, the different classification of nanocarriers based on lipids were listed below (Figure 2).

## Anocarriers Based on Polymers Branches

In general, polymers are made up of multiple linear and branched co-polymers or cross-linked polymer networks. The physical and chemical changes in the polymer in response to external factors like pH and temperature provide them with distinctive features (10). Polymer-based nanocarriers have tree-like structures and consist of hyperbranched polymers, dendrigrafts, dendrons and dendrimers. Each of these four classes reflects the structural features of these complex macromolecular architectures (46).

### Polymer Nanoparticles

Polymer nanoparticles, which were invented in the 1970s, are more stable than liposomes and their preparation methods are easy to reproduce. In addition, the controlled release properties of polymer nanoparticles are easier to control and surface modification is convenient (47). In general, polymer nanoparticles can be divided into two categories: nanospheres and nano microcapsules. Nanospheres are “matrix type,” in which drugs are dispersed throughout the matrix, while microcapsules are “repository type,” in which drugs are present in a core surrounded by a polymer shell. In recent years, biodegradable nanoparticles have shown great potential as drug carriers due to their good biocompatibility.

However, the encapsulation rate of polymer nanoparticles is low, and due to the high molecular weight of polymer, polymer nanoparticles can easily induce immune response. To overcome these shortcomings, a new carrier system emerges out which is known as lipid polymer hybrid nanoparticles (LPNs). Clearly, the polymer controls the drug release and the lipid increases the loading efficiency as well as permeation. Thus, LPNs have good potential to enhance physical stability and biocompatibility (48). Zhang et al. (49) constructed a polymer-lipid hybrid nanoparticle in which polyetaxel is encapsulated in the polymer core, which is encapsulated in a monolayer of lipids. Compared with pure polymer nanoparticles (37%), this system has a higher encapsulation rate (59%). In addition, the lipid-controlled release barrier on the surface of the system inhibits the burst release effect, and it takes 20 h for the mixed nanoparticles to release 50% of the drug, compared to only 7 h for the pure polymer nanoparticles. LPNs system has been explored for the oral delivery of several thrombolytic agents. Previous reports demonstrated that chitosan and lipid nanoparticles increased the oral bioavailability of heparin (50). Khan (51) reported that cisplatin loaded lipid-polymer hybrid nanoparticles was an effective delivery system for controlled drug delivery at tumor sites, having promise as a platform for controlled delivery of cisplatin in cancer therapy.

### Polymer Micelles

Polymer micelles are a class of nano-colloids, which can be formed by self-assembly of amphiphilic block copolymers in aqueous solution (52). In polymer micelles, hydrophobic drugs are encapsulated in the hydrophobic core, while the hydrophilic shell plays a role in maintaining particle stability, which makes it suitable for intravenous injection (53). It usually presents a core-shell structure, and its particle size is generally distributed in the range of 5–100 nm (54). Unlike nanoparticles, polymer micelles are typically characterized by critical micellar concentration (CMC). Therefore, when the concentration is diluted below the CMC, the micelles disassemble into free unimers (55). Nonetheless, polymer micelles mainly have low CMC values, which render them relatively insensitive to dilution, thus leading to enhanced circulation times. According to research results, polymer micelles have been able to successfully deliver a variety of hydrophobic drugs and DNA and can be functionalized by many different ligands (56, 57). In addition, compared to non-polymer-stabilized micelles, polymer-stabilized micelles can reduce rapid drug clearance *via* strengthening of the micellar structure and increase in the available drug amount in plasma, thus broadening pharmaceutical applications of micelles (58).

### Polymer Vesicles

Polymer vesicles (or polymersomes) are also similar to “storehouse” systems, but have different properties from polymer nano-microcapsules. Polymer vesicle membranes are composed of special amphiphilic block copolymers, biomimetic analogs of natural phospholipids, ranging in size from tens of nanometers to tens of microns, and can be well customized to the desired size (59). The hydrophobic segments of copolymers gather together to reduce contact with water, while the hydrophilic groups are distributed on the outer side of the membrane, thus forming a typical bilayer membrane structure similar to that of liposomes (60). Therefore, similar to liposomes, polymer vesicles can encapsulate hydrophobic substances in the membrane, while sub-encapsulating hydrophilic groups in the water-based core which are more complicated than polymer micelles with a simple core–shell nanostructure. Recently, polymer vesicles have attracted increasing attention due to their low permeability, superior stability and toughness (61). Polymer vesicles evolved from a simple “hollow sphere” to “smart” nanocarrier which were responsive to external stimuli (62), and presented significant advances for promising applications in biomedical field, including drug delivery, gene therapy, magnetic resonance imaging, theranostics over the past decades (59).

### Dendrimers

**Figure 3:**
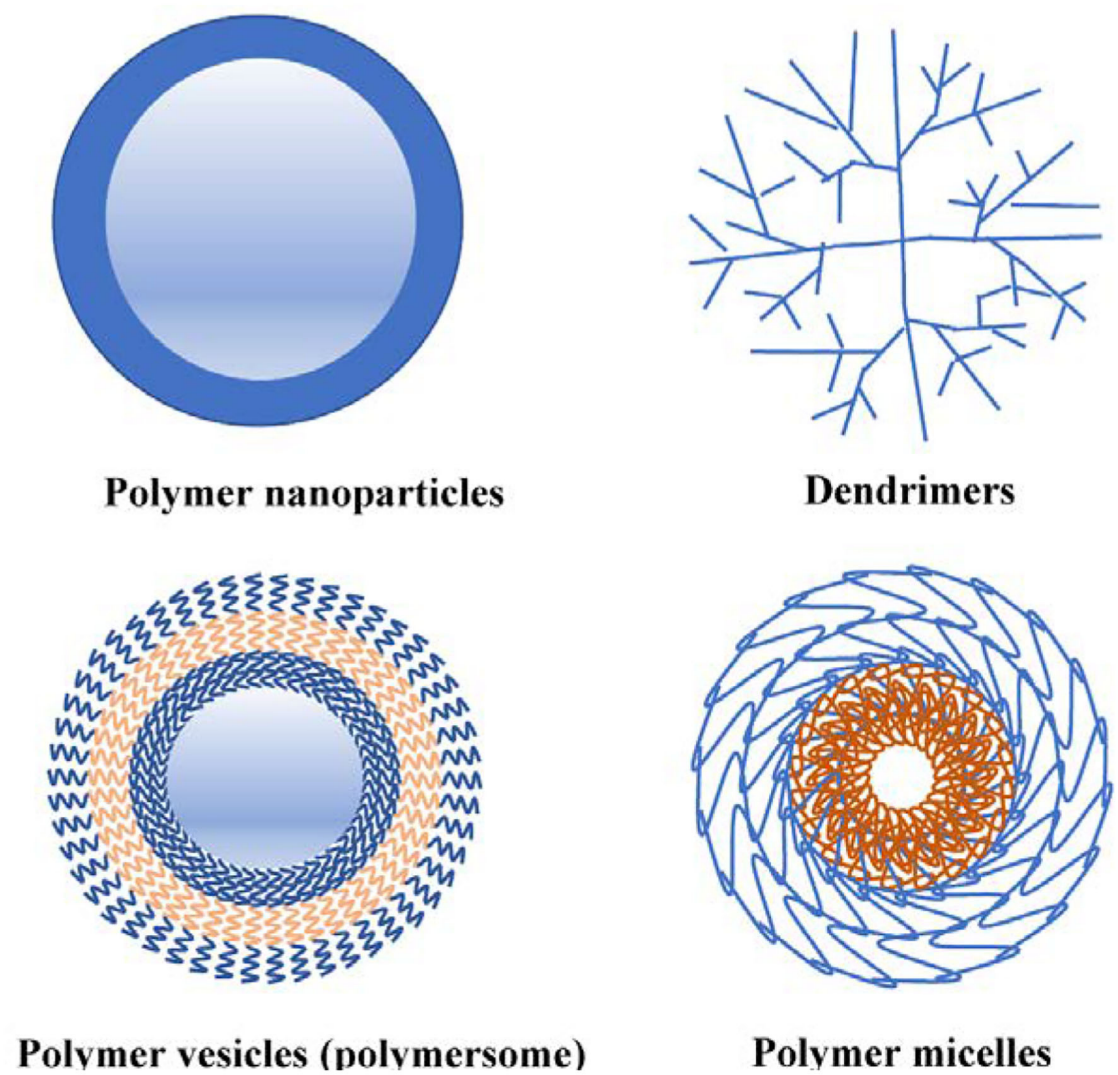
Different types of polymer-based nanocarriers.

Dendrimers are highly branched polymer molecules consisting of a core and branches connected around the core, typically between 5 and 20 nm (<100 nm) in size (63). Due to the particularity of dendritic molecules, dendritic molecules, as nanocarriers, have the advantages of high stability, easy size control and easy surface functionalization (64) Dendrites have been reported to improve the solubility of refractory anticancer drugs through ionic interactions, hydrogen bonding and hydrophobic interactions. In addition, dendritic branches have multiple sites for binding multiple drugs or targeting groups (65). Compared to liposomes, dendrimers present some advantages. dendrimers can be covalently bound to drugs, which are stable (63). However, once liposomes are systems composed of several components, drugs cannot be covalently attached to their outer surface and are difficult to stabilize (66). The aforementioned different type of polymer-based nanocarriers were bellowed (Figure 3).

## Application of Nanocarrier in the Delivery of Pharmaceuticals

Nanoparticle drug delivery systems have been used in the clinic since the early 1990's. The past decade has witnessed tremendous advances in the field of nanoparticle engineering technologies. Recently, nanotechnology-based drug delivery systems such as nanoparticles (67), polymer micelles (68, 69), polymer vesicles (70), dendrimer (71), liposomes (72), SLN (73), NLC (74) and nanoemulsion (75) have been demonstrated to be excellent platforms for drug delivery to address the oral delivery challenges. Among them, liposomes most commonly was used in nanoformulation for drug delivery of a wide range (76). Liposomes have been widely used to develop drug delivery agents with clinical potential due to their biocompatibility, ease of modification and ability to reduce drug side effects. Because liposomes can simultaneously contain hydrophobic substances in bilayer and hydrophilic substances in core, and cationic liposomes can bind therapeutic genes, liposomes based on the combination of two drugs, drug and metal, and drug and therapeutic genes have been reported (77). Additionally, liposomes have also been explored as “chemical containers,” designed to serve as nanoscale reaction vessels for remotely controlled reactions (78). It has been demonstrated that liposome-based nanocarrier has effective therapeutics on anti-cancers, anti-hyperlipidemics, anti-inflammatories, antimalarials, anti-parasitics (79). Despite significant advances in medical science and technology, cancer remains a disease with limited treatment approaches. The combination of anti-tumor drugs based on liposome can improve the anticancer effect, anti-proliferation activity, pro-apoptotic activity and cytotoxicity of the drugs. Meanwhile, the preparation can reduce the systemic toxicity of drugs. In addition, the co-delivery of chemotherapy drugs using liposomes can destroy drug resistance in cancer cells (77). It has been reported that encapsulation of etoposide with positively charged monolsomes can enhance its antitumor activity and reduce etoposide effects (such as bone marrow suppression) (80).

Generally speaking, the driving force of nanocarrier in tumor tissues was based on enhanced permeability and retention (EPR) mechanism (81, 82). Utilizing the passive mechanisms of EPR still remains a critical design parameter of nanocarrier delivery to tumors, however, inefficient drug release and uptake by the endosomes can limit their therapeutic efficacy (83). Correspondingly, it does not necessarily ensure delivery of the cargo within the tumor cell *via* EPR mechanism facilitates (84). Hence, directing nanocarrier surface-modified with ligand motifs specific to such receptors provides a promising way to exploit the receptor-mediated active endocytosis mechanisms to achieve intracellular delivery of the nanocarrier. This mechanism of “active targeting” has been investigated for various receptors. Recently, converging lines of evidence suggest that cell surface receptor, long non-coding RNAs (lncRNAs) and cytokines such as integrins, folate, growth factors, which are known to express on disease cells and their microenvironments, were explored for developing counter marker functionalized drug carrier to recognize the target disease cells (74, 85, 86). Especially in antitumor treatment, cell surface receptor or tumor necrosis factor such as epidermal growth factor receptor (EGFR) (87, 88) and tumor necrosis factor-related apoptosis-inducing ligand (TRAIL) (89) has been successfully designed as a target or mediator of ligand-nanoparticle or ligand-drug conjugates for active drug delivery.

## Application of Lipid-Based Vehicles for Nanoencapsulation of Food Industry

Currently, in the preparation of safe food, with superior nutritional and sensory characteristics, and multiple health benefits, nanofoods based on the application of nanotechnologies was newly proposed. Hence, for delivering functional ingredients and nutraceuticals into body cells and protect them against detrimental environmental and food process factors, food bioactive ingredients, including phenolic compounds, antioxidants, natural food colorants, antimicrobial agents, essential oils, minerals, flavors, essential fatty acids, and vitamins, could be placed within nanocarriers such as nanoemulsions, NLCs, SLNs, micro-emulsions and nano-liposomes so that a controlled/targeted release could be achieved (90–94). Commonly, food lipids used to encapsulate nutrients and functional components have been proven to be effective in protecting, controlling the release, and enhancing the action of bioactive compounds (95). Especially, the stability and absorption of polyunsaturated fatty acids, which are easily affected by heat and light, can be improved by wrapping them with liposomes. Nanoencapsulation of essential oils offers numerous advantages such as ease of handling, stability, protection against oxidation, enhanced distribution, solubility, controlled release, with less or no adverse effect on organoleptic properties of applicable food items with enhanced bioavailability (96). Bai et al. (97) prepared coix seed oil liposomes, which improved the stability of coix seed oil, improved the solubility of coix seed oil in water and significantly promoted its absorption in intestine. When using liposomes to wrap antioxidant components, liposomes can not only improve the resistance of antioxidant to adverse environment, but also enhance its antioxidant effect to a certain extent. According to previous reports, resveratrol liposomes show better ROS quenching ability than resveratrol, and can exert better antioxidant activity in cells (98). Apart from the attribute of saving bioactive food ingredients from environmental damage, it is also believed that one of the ways to disguise their displeasure attributes is through nanoencapsulation, especially active ingredients with undesirable flavor or properties that cannot be applied directly to food. Even though oleuropein from olive has numerous benefits, but it cannot be used for food fortification because of its presence in enzymatic browning reactions, bitter taste and irregularly absorbed in the gastrointestinal tract due to antioxidant activity. It has been demonstrated that adding olive leaf extract-loaded nanostructured lipid carriers powder as the source rich of oleuropein to sauce, could improve the undesirable appearance, color, odor, and flavor of the extract powder (polyphenols such as oleuropein) and also enjoy high durability of antioxidant properties of the polyphenols existing in the nanocarrier (99). It also reported that lipid-based nanocarriers stabilized by hydrophobically modified starch or sucrose stearate for the delivery of lutein could be appropriate for hydrophobic bioactive compounds in nutraceutical beverages (100).

Besides, lipid-based nanocarrier can also be used for solubilization of adjuvant or flavoring agent in food industry and improve its processing sensitivity. Lee et al. (101) has demonstrated the influences of carrier lipids (medium-chain triglyceride, coconut, olive, and soybean oil, and cocoa butter) on the physical stability of β-caryophellene-loaded lipid nanocarriers when they were exposed to various food processing conditions including thermal, freeze-thaw, and salt treatments, and revealed that the β-caryophellene-loaded lipid nanocarriers with medium-chain triglyceride oil could greatly benefit from application to various food processing.

## Application of Polymer-Based Nano-Carriers in Food Processing

Due to its excellent encapsulation performance, sustained release ability, as well as efficient biocompatibility and biodegradability, polymer-based nanocarriers are also widely used for embedding food functional factors, nutrients and additives (listed in Table 1). Generally speaking, there are two type of polymer material, including natural and synthetic polymer nanoparticles, were used as polymer-based nanocarrier. Compared with synthetic polymers such as polylactide–polyglycolide copolymers, polyacrylates and polycaprolactones (PCL), polylactic acid (PLA), poly (lactic-co-glycolic acid) (PLGA) which are often used in nanoparticle synthesis (113–115), natural polymers polysaccharides are more concerned by food researchers because of their good safety. However, in most cases, polysaccharides need to be modified to have the desired nanocervative function. Modified-polysaccharides-based wall materials mainly include modified chitosan, modified starch and other materials. As for chitosan concern, it is mucoadhesive and soluble only at acidic pH. Hence, chemical modification of chitosan is being carried out to enhance its mucoadhesive properties. Chitosan derivatives like trimethyl chitosan (TMC), thiolated chitosan, chitosan-ethylenediaminetetraacetic acid, etc. have showed improved solubility and mucoadhesive properties (116). It is reported that the modified chitosan wall material has good solubility and film forming properties. Peng et al. (117) synthesized water-soluble methoxy poly (ethylene glycol)-graft-chitosan and applied it to prepare nano-microcapsules encapsulated in seaweed oil. The results showed that such modified chitosan nano-microcapsules had a high encapsulation rate on seaweed oil and could avoid the effects of ultraviolet light and heat on the quality of seaweed oil. Rusli et al. (118) successfully embedded fish oil vulnerable to oxidative damage by taking the product of maillard reaction between protein and carbohydrate as the wall material of nano-microcapsules. In particular, the wall material itself has some antioxidant activity, which can better protect fish oil composition (119). Notably, natural plant- and microbial-derived polysaccharides, such as soy soluble polysaccharide (106), modified pectin (107), can also be used as nanocarriers.

**Table 1:** Summary of phytochemicals encapsulating polymer nanocarriers in the food industry.

**Nanocarrier formulation**	**Drug payload**	**Function**	**References**
Acetylated debranched starch micelles	Curcumin	Increased solubility and controlled-release	(102)
Acetylated polysaccharide from *Sterculia striata*	Amphotericin B (AMB)	Increasing AMB solubility and decreasing toxicity	(103)
Ovalbumin-propylene glycol alginate nanocarriers	Anthocyanins	Enhance the resistance of anthocyanin to environmental stress in food process	(104)
Ovalbumin-carboxymethylcellulose nanocomplexes	Resveratrol	Enhance the photostability and bioaccessibility	(105)
Soy soluble polysaccharide	Curcumin	Enhanced water solubility, stability, and bioaccessibility of hydrophobic bioactives	(106)
Pectin-decorated selenium nanoparticles	Curcumin	Enhanced water solubility, stability, and *in vitro* bioactivities	(107)
Chitosan nanoparticle	Cinnamon essential oil	Excellent antimicrobial and antioxidant property for the pork during refrigerated storage	(108)
Modified starch nanoemulsion	Clove essential oil	Prolonged antibacterial activities against Gram positive bacterial strains	(109)
Glycosylated casein	Epigallocatechin-3-gallate	Strong encapsulating and retaining capacity	(110)
Oligochitosan-modified apo-red bean seed ferritin	rutin	High thermal stability in rutin	(111)
11S quinoa seed protein	Betanin	Increase protein solubility exert the betanin controlled delivery protected betanin from light and oxygen stress	(112)

Additionally, some type of food-derived proteins with special structures can be used as nanocarriers (120). Protein nanocages, usually assembled from multiple subunits in a high symmetry, are three-dimensional shell-like containers, which possess an intrinsic homogeneous chamber that is segregated from the bulk environment by protein walls (121). Structurally, natural nanocages include ferritin, heat shock protein, DNA-binding protein, and encapsuling (121–123) with a modifiable exterior surface. Previous research demonstrated that applications of chitosan and transglutaminase in protein modification will improve ferritin functionalization as a nanocarrier for food bioactive molecules (111). It has been reported that betanin loaded 11S quinoa seed protein nanovehicle could exert the betanin controlled delivery for pharmaceutical and nutraceutical food products (112). Additionally, meat-, milk-, egg-, and silk-based protein, as well as plant-based proteins, such as zein proteins, soy proteins, pea proteins have been reported as possible carriers for bioactive compounds (120, 124). It has been demonstrated that casein and glycosylated casein display strong encapsulating and retaining capacity to epigallocatechin-3-gallate (EGCG), effectively protect EGCG from degradation in alkaline pH and displayed a slow and sustained release in intestinal fluid. Therefore, glycosylated casein could be used as a promising and effective nanocarrier for EGCG (110).

The application of polymer-based nanocapsule to plant essential oil is a hot spot in the field of food science. Nano microcapsules containing essential oil can play a role in food flavoring, anti-corrosion and anti-oxidation. The essential oil in traditional spices is attached to the carrier of wood fiber, which is difficult to be fully utilized. However, the use of nano-microcapsules to wrap the essential oil in spices can reduce volatilization loss, inhibit quality deterioration and facilitate storage and transportation (125). Bai et al. (126) used chitosan/polycaprolactone nanoparticles as wall materials to prepare tea tree essential oil microcapsules, which showed higher protection against volatilization and exhibits broader anti-bacterial spectrum. Moreover, polymer-coated essential oils are good for keeping fresh food. Wu et al. (127) prepared nano microcapsules of citrus essential oil using chitosan and sodium tripolyphosphate as wall materials, and studied its film-forming ability and fresh-keeping function for fish. The results showed that citrus essential oil nanocapsules could significantly prolong the shelf life of marine fish and have a broad prospect of application in the field of marine product preservation. Hu et al. (108) reported that chitosan nanoparticles loaded with cinnamon essential oil exhibited the excellent antimicrobial and antioxidant property for the pork during refrigerated storage. In addition, polymer nanocarriers solve the problems of limited application of hydrophobic food functional components in aqueous food, such as poor water solubility, poor stability and low bioavailability. Liu et al. (102) reported that acetylated debranched starch micelles could be designed as a promising nanocarrier for controlled-release of curcumin in simulated digestive fluid.

## Safety of Nanocarriers

Although a large number of research reports that lipid-based and polymer-based nanocarriers are expected to be applied to the pharmaceuticals and food industry, however, safety should not be underestimated. It is hypothesized that nanocarriers having properties such as nano-size, high surface to volume ratio, and infuse efficiently from the intestinal barrier to circulation may cause toxicity since they differ from bulk material (128). Nanoparticles may exert toxic effects such as oxidative stress, inflammation and DNA damage in animals, after entering the human body, contacting with cells and producing toxic effects (129, 130). Recently, increasing evidences have demonstrated that the induction of autophagy by nanoparticles can also cause nanotoxicity (131, 132). Additionally, concern about penetration of biopersistent solid nanoparticles into the skin which could lead to damage to viable epidermal cells or accumulation in secondary organs following biodistribution was also in the spotlight (133, 134). Fortunately, there are increasing reports on the safety evaluation of nanocarriers. A growing body of evidence suggests that certain bioactive substance-loaded nanocarriers are efficient and safe formulations that can be used in food or medicine. In assessing the gel properties, pharmacokinetics, morphology, anticancer activity and immunohistopathology after thermosensitive irinotecan-encapsulated solid lipid nanoparticles (SLN) rectal administration to tumor-bearing mice, it was easily administered to the anus, gelling rapidly and strongly after rectal administration, inducing no damage to the rat rectum and no body weight loss in tumor-bearing mice (135). Futhermore, mutiple nanocarrier, including bio-nanocapsule-liposome nanocarrier (136), poly{(benzyl-L-aspartate)-co-[N-(3-aminopropyl) imidazole-L-aspartamide]}-poly(ethylene glycol) (137), Polysaccharide-based nanocarriers (138), (139), Poly(aspartic acid-graft-imidazole)-poly(ethylene glycol) [P(Asp-g-Im)-PEG] (140), amphiphilic copolymer of poly(epsilon-caprolactone)-b-poly(ethylene glycol)-b- poly(epsilon-caprolactone) (141), were demonstrated to be safety *in vivo* in treatment cancer, inflammatory bowel disease. Additionally, some material was demonstrated to be safety such as starch-based nanoparticles (139). While a vast array of nanocarrier is under development, many of which are undergoing advanced clinical trials. However, relatively few have achieved full translation to clinical practice. This slow uptake may be due, in part, to the need for a rigorous demonstration of safety in these new nanotechnologies.

For food safety as regards, although the safety (or the safety dose) of most food materials has been established, the safety of food obtained through nano processing needs to be further explored. It has been demonstrated that ingestion biodegradable polymers chitosan-sodium alginate-oleic acid based nano-carrier loaded with lutein (LNCs) with dose of 10 mg/kg body weight was demonstrated to revealed no mortality with no morphological and clinical changes in rats (128). However, there are few safety-evaluation studies on nanocarriers used in food under strict scrutiny. Therefore, the application of effective nanocarriers in the real food industry still needs a large space for safety evaluation.

## Conclusion and Future Prospect

Owing to marked progress in the understanding of nanoformulation discoveries and petentional applications, substantial efforts done for successful management of lipid-based or polymer-based nanocarrier with least adverse effects as medicine or as an protectant, enhancer or improver for bioactive ingredient in the food industry. In terms of nanomedicine, mutiple researches have focused on targeted delivery of anticancer drugs. Therefore, the wider use of nanocarriers in medicine should be covered. Moreover, nanocarrier used in nanomedicine may transform into possibly higher toxicity and immunogenicity as well, thus increasing manufacturing cost and unexpected risks. Hence, designing new nanoformulations require sufficient proofs to demonstrate that nanoformulation by certain nanocarrier is clinically more active, adequately stable and cost-effective. Considering food application respect, low-cost, efficient, safety and edible nanocarrier formulations has a broad application prospect in food preservation, preservative, functional ingredient enhancement and flavor ingredient sustained release, which is also the urgent demand in food industry. Therefore, effective, safe and non-toxic nanomaterials for food use require multiple experimental data and proofs. To conclude, nanocarriers have broad prospects in food and medicine, and should receive more attention, especially for safe phytochemical.

## Author Contributions

HL and QC conceived the review topics. HL wrote the initial draft. SZ revised the manuscript. JW and QC revised and supervised the entire work. All authors read and approved the submitted version.

## Funding

This research was funded by the Science and Technology Project of Yunnan province (No. 202002AA1000056-3).

## Conflict of Interest

The authors declare that the research was conducted in the absence of any commercial or financial relationships that could be construed as a potential conflict of interest.

## Publisher's Note

All claims expressed in this article are solely those of the authors and do not necessarily represent those of their affiliated organizations, or those of the publisher, the editors and the reviewers. Any product that may be evaluated in this article, or claim that may be made by its manufacturer, is not guaranteed or endorsed by the publisher.
